# A study of inflammatory biomarkers in crystalline silica exposed rock drillers

**DOI:** 10.1007/s00420-024-02070-2

**Published:** 2024-05-04

**Authors:** Dag G Ellingsen, Liv Ingunn Bjoner Sikkeland, May Britt Lund, Nils Petter Skaugset, Bente Ulvestad

**Affiliations:** 1 National Institute of Occupational Health, Pb 5330, Majorstuen, Oslo, N-0304 Norway; 2 Faculty of Medicine, University of Oslo, Oslo, Norway; 3 Department of Respiratory Medicine, Oslo University Hospital, Oslo, Norway

**Keywords:** Biomarkers, Matrix metalloproteinase 12, Inflammation, Crystalline silica

## Abstract

**Background:**

Crystalline silica (CS) exposure can cause serious lung disease in humans, but mechanisms of pulmonary toxicity have not been completely elucidated.

**Aims:**

To assess pro-inflammatory and anti-inflammatory biomarkers and biomarkers related to the development of chronic obstructive pulmonary disease and fibrosis in serum of rock drillers exposed to CS.

**Methods:**

Rock drillers (*N* = 123) exposed to CS and non-specified particulate matter (PM) were compared to 48 referents without current or past exposure to PM in a cross-sectional study.

**Results:**

The rock drillers had been exposed to CS for 10.7 years on average. Geometric mean (GM) current exposure was estimated to 36 µg/m^3^. Their GM concentration of matrix metalloproteinase 12 (MMP-12) was significantly higher (16 vs. 13 ng/L; *p* = 0.04), while interleukin (IL) 6 and IL-8 were significantly lower compared to the referents. Also pentraxin 3 was significantly lower (3558 vs. 4592 ng/L; *p* = 0.01) in the rock drillers. A dose-response relationship was observed between cumulative exposure to CS and MMP-12, the highest exposed subgroup having significantly higher MMP-12 concentrations than the referents.

**Conclusion:**

Exposure to CS may increase circulating MMP-12 concentrations in a dose-response related fashion. The results may also suggest a down-regulation of pro-inflammatory pathways.

## Introduction

Rock drillers in Norway operate different types of rock drilling equipment that, during use, generate airborne dust containing crystalline silica (CS) when present in the rock. Inhalation of CS is a well-known risk factor for silicosis and lung cancer, but also chronic obstructive pulmonary disease (COPD) has been associated with such exposure (Hnizdo and Vallyathan 2003; Bergdahl et al. 2004; Leung et al. 2012; Möhner et al. 2013; Brüske et al. 2014; Tavakol et al. 2017).

The mechanisms of pulmonary CS toxicity have not been completely elucidated. Proposed mechanisms include destruction of pulmonary surfactant or the modification of silica toxicity by surfactant covering the particles, the ability of CS to form free radicals, surface chemistry, the ability to increase lysosomal permeability and the role of silica to induce macrophage immune dysfunction (Hamilton et al. Jr 2008; Pavan et al. 2019).

Biomarker studies of CS exposed humans have focused on inflammation, pneumoproteins and other types of biomarkers, and the results have often been inconsistent (BlancoPérez et al. 2021; Peruzzi et al. 2022; Călutu et al. 2023). This may partly be related to the selection of subjects. Some have studied patients with silicosis, while others have included subjects with poor pulmonary function and ongoing exposure. It is possible that both ongoing exposure and pulmonary pathological alterations associated with exposure may cause changes in biomarker concentrations.

Recent studies in mice have indicated mechanisms involving a shift in pulmonary macrophage polarization. A more “proinflammatory” M1 macrophage phenotype early after CS administration and a more “anti-inflammatory” M2 macrophage phenotype later in the chain of pathological events leading to fibrosis have been shown (Xiang et al. 2016; Zhao et al. 2020). Zhao et al. (2020) reported increased destruction of the alveolar integrity as an early event after exposure followed by the development of fibrosis at a later stage. They further reported lung tissue M1 macrophages to increase up to 14 days post-exposure and a decrease thereafter, while a substantial increase of M2 macrophages occurred from day 28 to 56 post-exposure in bronchoalveolar lung fluid (BALF) (Zhao et al. 2020). A more pronounced increase of interstitial M2 macrophages was reported from day 42 post-exposure in that study. The mRNA expression of the pro-inflammatory cytokines tumor necrosis factor (TNF), interleukin (IL) 6, and IL-1β was significantly higher during the first 14 to 28 days post-exposure and significantly lower than the controls thereafter (Zhao et al. 2020). Further, the anti-inflammatory IL-10 was significantly lower up to 7 days post-exposure and significantly higher thereafter compared to controls. It is also noteworthy that transforming growth factor beta (TGF-β), which is involved in bleomycin induced fibrosis (Wynn and Vannella 2016), was not altered post-exposure. However, the exposure was very high. Also, the mice were exposed once, in contrast to humans occupationally exposed for years. Similar studies have not been conducted in humans.

We have previously reported a decline in pulmonary function, mainly small airways dysfunction, in the absence of silicosis in rock drillers exposed to CS (Ulvestad et al. 2020). The rock drillers also had alterations in their serum levels of surfactant proteins (SP), especially SP-D (Ellingsen et al. 2022). The aim of the present work was to assess the impact of exposure on serum concentrations of pro- and anti-inflammatory biomarkers and other biomarkers assumed to be related to the development of fibrosis and macrophage polarization in the same rock drillers. Lung fibrosis and COPD are complex diseases, but it has been suggested that macrophage polarisation plays a key role in the pathophysiology of both. In this study it was hypothesized that altered macrophage polarisation may be involved when exposed to CS.

## Materials and methods

### Study population and design

Three large construction companies in Norway, engaged in outdoor rock drilling, were invited to participate in this cross-sectional study, and they all agreed. At the time of the study, the companies employed in total 140 male rock drillers, defined as working in the immediate vicinity of a drilling rig. These subjects were invited to participate. Four subjects declined participation, and 13 were no longer exposed at the time of the examinations and were therefore excluded. Thus, the study comprises 123 currently CS exposed subjects. Referents (*N* = 48), that according to our knowledge, had never been occupationally exposed to particulate matter (PM) or CS, were recruited among administrative personnel, typically foremen and other non-exposed heavy construction workers. They were working at the same construction sites as the rock drillers. None of the potential referents declined to participate.

### Job descriptions

Details on job descriptions and exposure have been published (Ulvestad et al. 2020). In short, rock drillers operate different types of moveable drilling equipment at work sites scattered throughout the country. Blasting leaders and guardrail installers work in the vicinity of the drilling operation. Six different job categories could be defined, depending on how the jobs were performed. The airborne exposure of everyone was closely connected to the job categories. This information was used for the calculation of cumulative exposure estimates.

### Clinical examinations

Medical examinations, including blood sampling, and recording of background data were carried out at the regional offices of the companies immediately after the end of a four days’ work week. Height and weight were measured for the calculation of body mass index (BMI). Previous and current jobs, occupational dust exposure, and the type of drilling equipment the rock drillers had used during their work career, were recorded. Smoking habits and medical history were also recorded. Spirometry, without the use of bronchodilators, was carried out as previously described (Ulvestad et al. 2020). Finally, venous blood samples were collected for the assessment of biomarkers.

### Blood sampling

Venous blood samples (10 mL) were collected in vacuum tubes without additives (Becton Dickinson and Company, Franklin Lakes, NJ, USA) and coagulated for 45 min before centrifugation (2000 g for 15 min). Serum was aliquoted into 4.0 mL NUNC^®^ polypropylene cryotubes (Thermo Fisher Scientific, Waltham, MA, USA), and thereafter frozen before long-term storage at -80 °C at the National Institute of Occupational Health (Oslo, Norway).

### Serum biomarker analysis

Protein levels of Chemokine ligand 17 (CCL17), CCL18, CCL22, matrix metalloproteinase (MMP) 9, Pentraxin 3 (ptx3) and TGF-β were measured using DuoSet ELISA kit obtained from R&D systems (Stillwater, MN, USA). MMP1 was measured using ProcartaPlex™ Multiplex Immunoassay (Affymetrix, eBiosciences, Japan) and MMP-12 using Luminex magnetic beads analysed on a BIO-PLEX (Biorad, Ca, USA). V-PLEX Proinflammatory Panel 1 Human Kit was used for quantitative determination of interferon gamma (IFN-γ), IL-1β, IL-2, IL-4, IL-6, IL-8, IL-10, IL-12p70, IL-13, and TNF (Meso Scale Diagnostics, USA). The expression of IL-1 β, IL-2, IL-4, 12p70 and IL-13 were low, and some of the values were below lower limit of detection (LOD). All analysis were performed according to manufactures instruction. A large proportion of IL-1β (*N* = 131/171) and IL-12p70 (*N* = 83/171) measurements were below LOD and were therefore not considered in the statistical analysis.

High sensitivity CRP in serum was determined at a clinical commercial laboratory (Unilabs, Oslo, Norway) using CardioPhase TM, Advia 2400 (Siemens Healthcare Diagnostics Inc., Tarrytown, NY, USA) with a method’s LOD of 0.1 mg/L.

### Occupational exposure measurements

Assessment of exposure to CS has been described previously (Ulvestad et al. 2020). Shortly, 23 selected construction sites of the three participating companies were surveyed between 2015 and 2018. Each job task was assessed by personal air sampling. Altogether, 98 and 93 air samples from the respirable and thoracic aerosol fraction, respectively, were collected, and the PM mass determined. Respirable α-quartz was measured by X-ray diffraction spectrometry in 67 air samples according to the silver filter NIOSH Method 7500 (NIOSH, 2003) with a Malvern Panalytical X’Pert^3^Powder diffractometer, equipped with a PIXcel^1D^ detector and an Empyrean X-ray tube (Malvern Panalytical B.V., Eindhoven, Netherlands). Procedures for the collection of air samples and determination of PM and α-quarz have been described (Ulvestad et al. 2020).

### Calculation of cumulative exposure

The concentrations measured in the air samples were used to calculate the mean exposure level of each of the six defined job categories. The information collected from the occupational history interview was used together with the arithmetic mean (AM) exposure for each year in each drilling job to calculate individual cumulative exposure to respirable crystalline silica by adding up each year of exposure. Further details on the calculations of cumulative exposure estimates have been published (Ulvestad et al. 2020).

### Statistics

The distributions of the variables were examined visually, Q-Q-plots were assessed, and skewness of the distributions were calculated. Data were log-transformed when the skewness exceeded 2.0. Geometric means (GM) are presented for those variables, while arithmetic means (AM) are presented otherwise. Minimum and maximum values (Min-Max) as well as 95% confidence intervals (95% CI) are also presented. Differences between two groups were assessed with the Studentʹs t-test, while analysis of variance (ANOVA) was used when more than two groups were compared. Chi-square test was used to assess differences between groups with respect to categorical variables, but Fisherʹs exact probability test was applied when expected figures were small (< 5). Associations between concentrations of biomarkers and several independent variables simultaneously were calculated with multiple linear regression analysis (backwards procedure). Independent variables included in the models were ever being exposed to crystalline silica (1/0), being a current smoker (1/0), age, BMI (kg/m^2^), current self-reported infection (1/0), self-reported use of asthma medication (1/0), and the time of blood sampling (in minutes after midnight). If ever being exposed to CS was associated with any of the biomarker concentrations, the variables cumulative exposure and current exposure were added separately to the model instead of ever being exposed. The multiple linear regression analysis was then performed among exposed rock drillers only. The rock drillers were finally stratified into three equally large groups according to cumulative exposure (low, medium, and high) for the visualization of dose-response associations adjusted for relevant confounders, using general linear models for adjustments. Two-tailed *p*-values < 0.05 were chosen as the level of statistical significance. The statistical data package SPSS, version 25.0 (IBM Corporation, Somers, NY, USA) was used for the statistical calculations.

## Results

The rock drillers and the referents were comparable with respect to age, current and former smoking habits, and BMI (Table 1). The rock drillers had been occupationally exposed to CS for an average of 10.7 years, and their current exposure level was estimated to a GM of 36 µg/m^3^. Blood samples were collected at similar times of the day among the exposed and referents (AM 693 vs. 695 min after midnight).

**Table 1:** Background characteristics and exposure data of the study participants

	Exposed (*N* = 123)		Referents (*N* = 48)		
	AM^†^	Min-Max	AM	Min-Max	*p*-value
Age (yrs.)	38.9	18–72	37.3	22–65	0.38
BMI (kg/m^2^)	27.0	20.2–41.7	26.2	21.0-41.4	0.23
Current smokers (%)^#^	20.3	-	25.0	-	0.51
Former smokers (%)^#^	20.3	-	14.6	-	0.39
Current infection (%)^#^	4.1	-	0	-	0.32
Use asthma medication (%)^#^	3.3	-	10.4	-	0.12
Years exposed	10.7	1–42	-	-	-
Cum quartz (yrs. x µg/m^3^) ^‡^	239	5-5885	-	-	-
Current quartz (µg/m^3^) ^‡^	36	12–440	-	-	-

^†^ arithmetic mean; ^#^ prevalence; ^‡^ geometric mean

Concentrations of MMP-12 were significantly higher among the rock drillers when compared to the referents (Table 2). Pentraxin 3 and IL-13 concentrations were significantly lower among the rock drillers. None of the other biomarkers differed significantly between the two groups. However, after adjustments for those variables presented in Table 3, the IL-6 and IL-8 concentrations were also statistically significantly lower in the rock drillers compared to the referents.

**Table 2:** Geometric mean (GM) concentrations of biomarkers among crystalline silica exposed rock drillers (*N* = 123) and referents (*N* = 48)

	Exposed		Referents		
	GM	Min-Max	GM	Min-Max	*p*-value
IL-2 (ng/L)	0.06	< DL^#^-0.92	0.08	< DL-0.86	0.38
IL-4 (ng/L)	0.02	< DL-0.40	0.02	< DL-0.49	0.43
IL-6 (ng/L)	0.59	0.08–2.7	0.77	0.25–215	0.13
IL-6_adjusted_	0.59	-	0.79	-	0.02
IL-8 (ng/L)	15	4.6–35	17	9.4–107	0.11
IL-8_adjusted_	14.8	-	16.9	-	0.048
IL-10 (ng/L)	0.23	0.06–3.1	0.23	0.08–1.4	0.96
IL-13 (ng/L)	0.93	< DL-6.7	1.2	< DL-3.7	0.03
IL-13_adjusted_	0.92	-	1.21	-	0.04
Interferon γ (ng/L)	4.9	1.4–117	4.9	1.6–58	1.00
TNF (ng/L)	2.0	1.1–26	2.0	1.2–3.5	0.53
CCL-17^†^ (µg/L)	0.20	0.03–0.56	0.20	0.05–0.58	1.00
CCL-18^†^ (µg/L)	47	12–108	44	10–90	0.44
CCL-22^†^ (µg/L)	0.42	0.18–0.74	0.45	0.26–0.72	0.12
MMP-1 (ng/L)	66	1.0–2950	98	5.0–893	0.15
MMP-9^†^ (µg/L)	680	191–2093	658	202–2119	0.74
MMP-12 (ng/L)	16	5.5–73	13	6.7–61	0.04
MMP-12_adjusted_	15.8	-	13.3	-	0.03
TGF-B^†^ (µg/L)	25	6.7–40	26	18–42	0.18
CRP (mg/L)	1.2	0.2–21.0	1.2	0.2–13	0.92
Pentraxin 3^†^ (ng/L)	3558	266–10432	4592	587–17364	0.01
Pentraxin 3_adjusted_	3568	-	4610	-	0.009

^†^ arithmetic mean; ^#^ detection limit

**Table 3:** Results from multiple linear regression analysis (backward procedure) including 123 currently crystalline silica exposed rock drillers and 48 referents

	β							
	Exp	Age	BMI	Smoke	Infection	Midnight	Asthmamed.	multiple *r*
IL-6^†^	-0.13^**^	-	0.02^****^	0.10^**^	0.23^*^	-	0.20^*^	0.40^****^
IL-8^†^	-0.06^**^	-	-	-	-	0.0002^*^		0.21^**^
IL-13^†^	-0.12^**^	-	-0.02^***^	-0.11^*^	-		-0.27^**^	0.30^***^
MMP-12^†^	0.07^**^	0.004^***^	-	0.12^***^	-	-		0.34^****^
Pentraxin 3^†^	-1042^***^	-	-127^***^	-745^*^	-	2.5^*^		0.34^****^

^****^p < 0.001; ^***^p < 0.01; ^**^p < 0.05; ^*^p < 0.10; ^†^ log-transformed

After considering potential confounders, ptx3 and the cytokines IL-6, IL-8 and IL-13 were negatively associated with ever being exposed as a rock driller, while MMP-12 was positively associated in the multiple linear regression analyses (Table 3). None of the other biomarkers were significantly associated with ever being exposed to CS. As IL-13 is known to be associated with asthma (Rael and Lockey 2011), subjects using asthma medication were removed from the regression analysis. IL-13 was then no longer associated with exposure, while the other biomarkers were. Thus, IL-13 was not assessed in the further analyses.

**Fig. 1:**
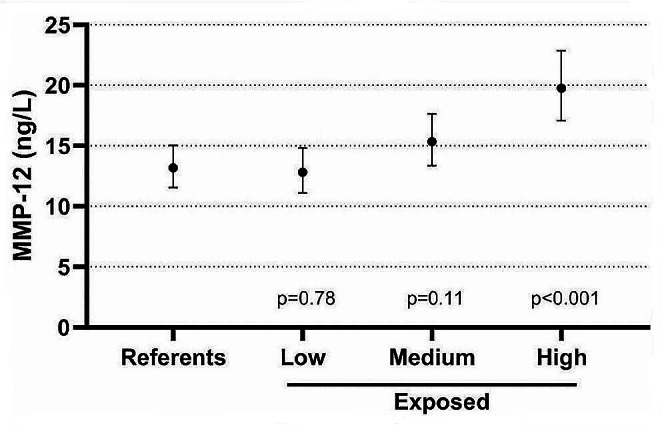
The geometric mean (and 95% CI) concentrations of serum MMP 12 among referents (*N* = 45) and rock drillers stratified into low (GM 44 µg/m^3^, min-max 5-114, *N* = 37), medium (GM 217 µg/m^3^, min-max 118–354, *N* = 41) and high (GM 1269 µg/m^3^, min-max 365–5885, *N* = 41) cumulative crystalline silica exposure adjusted for age and current smoking. *P*-values refer to comparisons with the referents

**Fig. 2:**
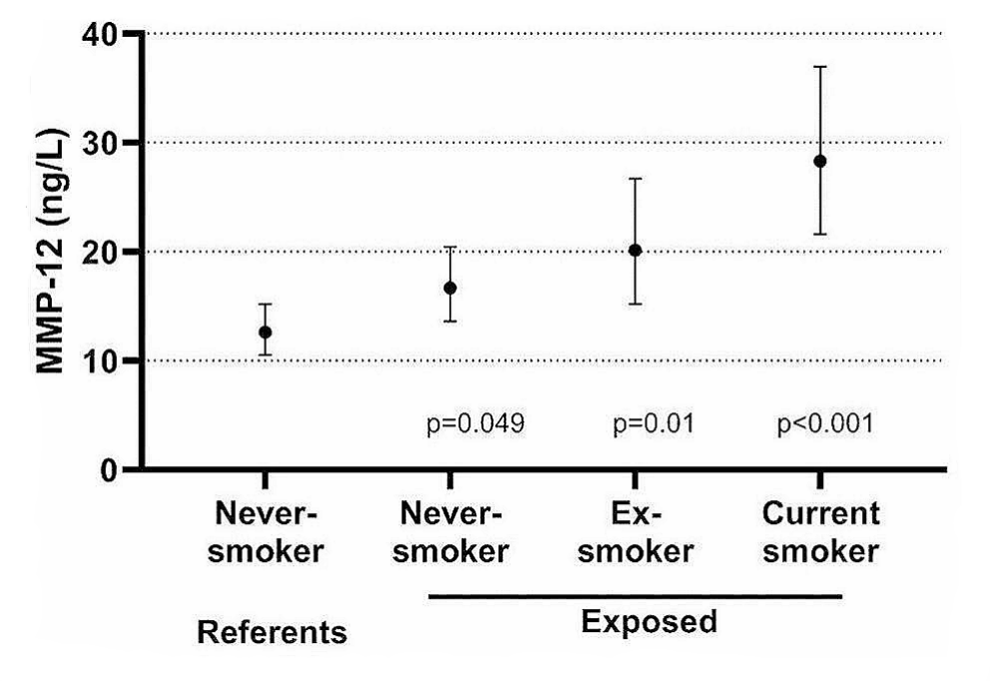
The geometric mean (and 95% CI) concentrations of serum MMP 12 among never-smoking referents (*N* = 26) and in the subgroup of rock drillers with highest cumulative silica exposure according to being a never-smoker (*N* = 19), ex-smoker (*N* = 11) and current smoker (*N* = 11). The concentrations are adjusted for age. *P*-values refer to comparisons with never-smoking referents

**Fig. 3:**
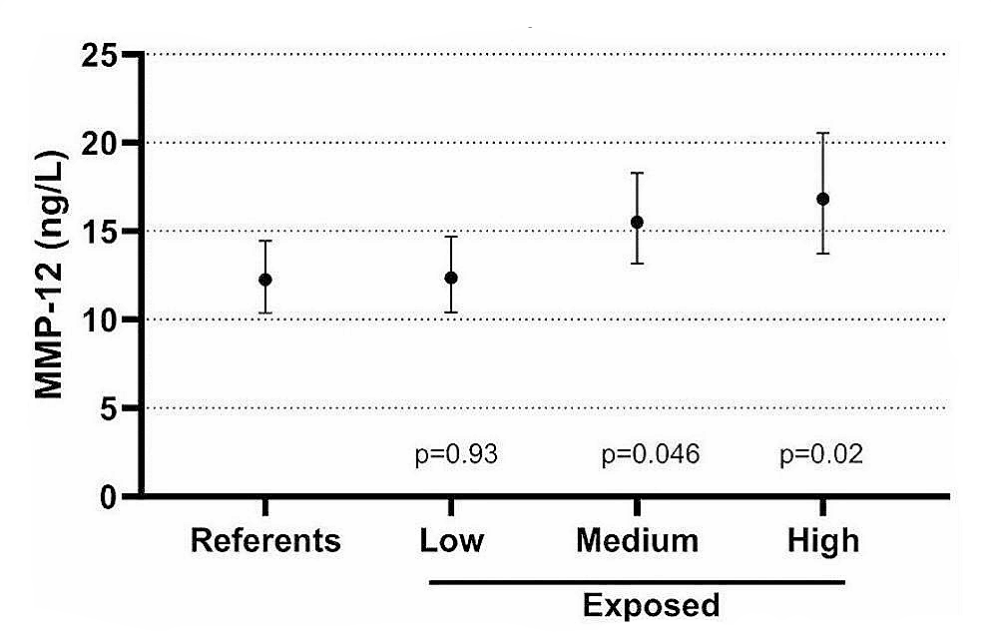
The geometric mean (and 95% CI) concentrations of serum MMP 12 among never-smoking referents (*N* = 23) and never-smoking rock drillers stratified into low (GM 50 µg/m^3^, min-max 11–114, *N* = 25), medium (GM 210 µg/m^3^, min-max 118–354, *N* = 28) and high (GM 1130 µg/m^3^, min-max 365–5885, *N* = 20) cumulative crystalline silica exposure adjusted for age. *P*-values refer to comparisons with the referents

Biomarkers that were associated with ever being exposed to CS, were assessed for their association with cumulative or current exposure. When considering only the exposed workers, there was a statistically significant association between MMP-12 and cumulative CS exposure. None of the other biomarkers were significantly associated with cumulative or current exposure dose estimates. Figure 1 shows the GM concentrations of MMP-12 among all referents and rock drillers stratified into three equally large groups according to cumulative exposure. The highest exposed subgroup had significantly higher MMP-12 than the referents. When stratifying the highest exposed subgroup according to smoking habits, never-smokers had the lowest and current smokers the highest MMP-12 concentrations, but all groups had significantly higher concentrations than never-smoking referents (Fig. 2). Figure 3 shows the association between cumulative exposure and MMP-12 among never-smokers.

No association was observed between MMP-9 and CS exposure, but current smokers (*N* = 37) had significantly (*p* = 0.006) higher concentrations than non-smokers (*N* = 129) (AM 811 µg/L; 95% CI 700–922) vs. AM 634 µg/L; 95% CI 575–694) adjusted for time of sampling.

**Fig. 4:**
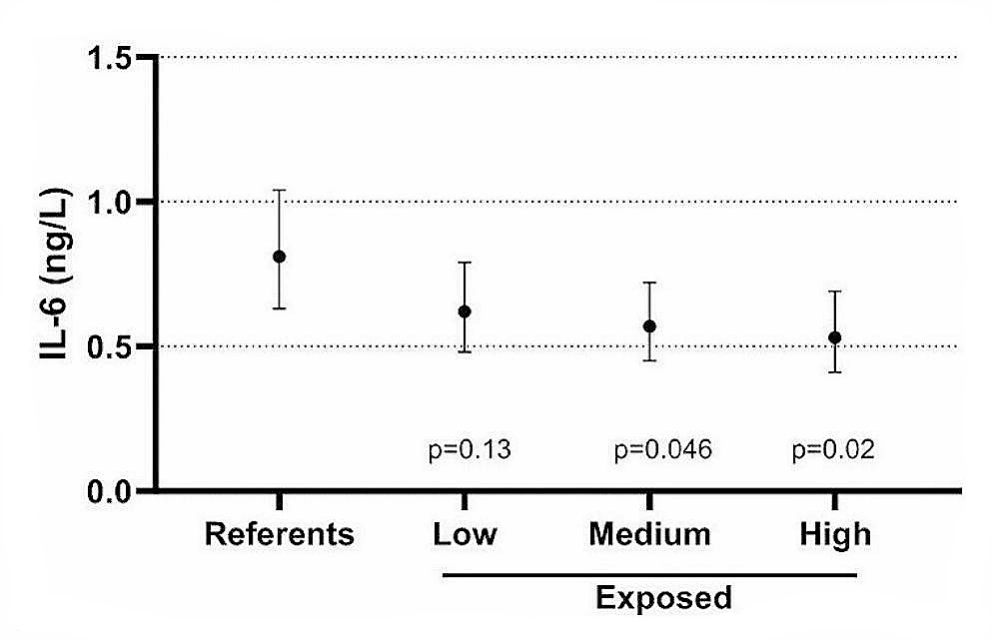
The geometric mean (and 95% CI) concentrations of serum IL-6 among non-smoking referents (*N* = 32) and non-smoking rock drillers stratified into low (GM 48 µg/m^3^, min-max 5-114, *N* = 31), medium (GM 216 µg/m^3^, min-max 118–354, *N* = 34) and high (GM 1144 µg/m^3^, min-max 365–5885, *N* = 20) cumulative crystalline silica exposure. The estimates are adjusted for infection, BMI and use of asthma medication. *P*-values refer to comparisons with the non-smoking referents

In a further analytical approach, the regression analysis was conducted without current smokers. No substantial changes occurred in the regression models. In a stratified analysis of non-smokers, there appeared to be a dose-response association between cumulative exposure to crystalline silica and IL-6 concentrations in non-smokers indicating lower IL-6 by increasing cumulative exposure (Fig. 4). No obvious dose-response pattern was found for IL-8 and ptx3.

Pulmonary function variables that were significantly lower in the rock drillers compared to the referents (Ulvestad et al. 2020) (maximal mid-expiratory flow % (MMEF%) and forced expiratory volume in one second/forced vital capacity ratio (FEV/FVC) were not associated with any of the biomarkers presented in Table 3. However, 34 rock drillers in the group with highest cumulative CS underwent pulmonary computer tomography (CT) (Ulvestad et al. 2020). Four of them were diagnosed with emphysema. Rock drillers with CT confirmed emphysema had significantly higher MMP-12 concentrations than those without emphysema on CT (GM 33.0 ng/L; 95% CI 24.2–45.0 vs. GM 19.7 ng/L; 95% CI 16.6–23.3, *p* = 0.03), while ptx3 concentrations were significantly lower (AM 1790 ng/L; 95% CI 200–3379 vs. AM 3919 ng/L; 95% CI 3167–4672, *p* = 0.048). No statistically significant differences were observed for IL-6 and IL-8 concentrations.

## Discussion

The rock drillers were all active workers and, to our knowledge, in good health at the time of the study. However, compared to the referents they had lower lung function, mainly related to small airways obstruction (Ulvestad et al. 2020), They also had altered surfactant protein concentrations in serum, particularly higher SP-D concentrations (Ellingsen et al. 2022). The main result of the present study was the higher MMP-12 concentrations among the rock drillers compared to the referents. Also, concentrations of pro-inflammatory cytokines (IL-6 and IL-8) and ptx3 were lower in the exposed group compared to the referents.

The GM concentration of MMP-12 was higher among the rock drillers when compared to the referents and there was an increase related to cumulative CS exposure. Further, four rock drillers with emphysema confirmed by pulmonary CT, had significantly higher MMP-12 concentrations than 34 rock drillers without emphysema. However, rock drillers with signs of small airways obstruction (MMEF%<70) had similar concentrations of MMP-12 to rock drillers with MMEF%≥70. Being a current smoker was also associated with higher MMP-12 concentrations.

To our knowledge, this is the first study that has reported an association between MMP-12 and CS exposure in humans. However, it is of interest that a recent study of rats reported a 14-fold increased gene expression of MMP-12 in the lungs after inhalation of high CS concentrations (Sager et al. 2020). A similar observation was reported in another recent study of rats (Cai et al. 2021). Thus, increased MMP-12 in the rock drillers appears to be a biologically plausible finding. In the present study, rock drillers with an estimated average CS exposure of 67 µg/m^3^ for 18.9 years, had statistically significantly higher MMP-12 concentrations than the referents.

MMP-12 is assumed to be synthesized in M2-polarized macrophages in humans (Gharib et al. 2018). MMP-12 has been extensively studied in animals exposed to other pollutants than CS. A study of mice exposed to PM_2.5_ by inhalation, showed increased M2 polarization of macrophages with a substantial upregulation of mRNA levels of MMP-12 (Jiang et al. 2020). Similar observations with increased expression of macrophage surface markers indicating higher percentage of M2 macrophages and increased expression of MMP-12 in the lung of mice exposed to cigarette smoke for 14 days was reported by Oliveira da Silva et al. (2017). A recent review points out that M1/M2 phenotypes exist as a spectrum that can be modified by cigarette smoke (Lugg et al. 2022).

MMP-12 is known to be involved in the process of elastin degradation leading to emphysema, although exact mechanisms are not known (Gharib et al. 2018). MMP-12 is also involved in certain types of pulmonary fibrotic disease (Abd-Elaziz et al. 2021; Chuliá-Peris et al. 2022). The role of MMP-12 in the development of emphysema is well established, which is in line with our observations of higher MMP-12 concentrations in the participants with emphysema confirmed by pulmonary CT (Churg et al. 2012; Chaudhuri et al. 2012).

Our data could suggest that increased MMP 12 could be part of a pathological process leading to emphysema. However, to what extent MMP-12 is involved in small airways remodeling in humans, another feature of COPD, is not known (Churg et al. 2012). The participants in our study with the lowest MMEF% predicted, indicating small airways alterations, did not have higher MMP-12 than participants with higher MMEF% predicted. However, knock out or inhibition of MMP-12 in animal models have been shown to prevent small airways remodeling (Churg et al. 2012). Further, microarray data of lung samples from patients with idiopathic pulmonary fibrosis has shown a significant upregulation of MMP-12 (Doni et al. 2021). A similar observation was reported in another recent study on rats (Cai et al. 2021).

We also observed significantly higher SP-D concentrations in these exposed rock drillers as previously reported (Ellingsen et al. 2022), SP-D has been shown to induce MMP-12 in human macrophages (Trask et al. 2001). Associations between SP-D and MMP-12 have hardly been studied and should be addressed in future studies.

It has been shown that MMP-12 increases during cigarette smoke exposure (Gharib et al. 2018), which is in accordance with our results. However, also never-smoking rock drillers had higher MMP-12 concentrations. This suggests that CS exposure, without concomitant cigarette smoke exposure, triggers biological pathways leading to increased MMP-12, and that current smoking will further increase MMP-12 levels. Ex-smokers, on the other hand, had lower MMP-12 levels than current smokers, suggesting that cessation of smoking is beneficial with respect to MMP-12 concentrations. Increased levels of MMP-9 have been observed in silicotic rat lungs (Scabilloni et al. 2005). This may indicate that exposure to CS was too low among the rock drillers for the induction of MMP-9. The observed increased levels of MMP-9 in alveolar macrophages in mice exposed long-term to cigarette smoke (Zhou et al. 2019), may be in accordance with our observations.

In the present study, we observed that the pro-inflammatory cytokines IL-6 and IL-8 were negatively associated with being exposed to CS. The IL-6 concentrations were also reduced in a dose-response pattern among non-smokers, with the lowest concentrations measured in the highest exposed subgroup. No such pattern was observed for IL-8. We have not found any previous studies of humans reporting similar observations. However, mice exposed to high concentrations of CS by intratracheal instillation, had significantly higher IL-6 concentrations up to 14 days post-exposure and significantly lower concentrations than the control mice from that time point up to the end of follow-up at day 56 (Zhao et al. 2020). That study also indicated that the IL-6 concentrations changed simultaneously with a shift in alveolar macrophage polarization from a more pro-inflammatory M1 phenotype up to day 14 post-exposure to a more M2 phenotype thereafter. Alveolar macrophages collected from the bronchoalveolar fluid of healthy current smokers had a clear M2-related gene expression profile compared to healthy non-smokers. A 2.2 fold lower expression of IL-6 was observed among the smokers although the difference did not quite attain statistical significance, while IL-8 was not measured. Pulmonary function was similar in the two groups (Shaykhiev et al. 2009). Another study showed significantly lower IL-6 concentrations released from alveolar macrophages collected from smokers compared to non-smokers after stimulation with the pro-inflammatory lipopolysaccharide (McCrea et al. 1994).

The pentraxins CRP and ptx3 were measured in the present study. While serum CRP concentrations were low and similar in the two groups, the ptx3 concentrations were significantly lower in the rock drillers as compared to the referents. This has to our knowledge not been reported in CS exposed workers previously. Pentraxin 3 is synthesized by a variety of cell types, e.g. fibroblasts, monocytes/macrophages, lung alveolar epithelial cells (Chang et al. 2022). Pentraxin 3 synthesis is stimulated by the pro-inflammatory cytokines IL-1β and TNF (Magrini et al. 2016). Pentraxin 3 has a multitude of biological roles, such as humoral innate immunity, microbial defense, regulation of inflammation and tissue remodeling and repair (Magrini et al. 2016). The role of ptx3 in tissue remodeling is mainly related to turnover of fibrin-rich deposits at wound sites, and like MMP-12, ptx3 is also considered to have a role in the development of pulmonary fibrosis (Doni et al. 2021). Microarray analysis performed on human lung samples collected from patients with idiopathic pulmonary fibrosis showed a substantial downregulation of the ptx3 expression (Doni et al. 2021). No such data exists, to our knowledge, for human pulmonary silicosis, but this should be elucidated in future studies.

The biomarkers that differed between the groups were all measured in serum, which not necessarily reflect alterations in the lungs of the participants. We also observed changes which may be related to the development of fibrosis, but spirometry and partly pulmonary CT of the participants, did not reveal any signs of fibrosis, but the rock drillers had alterations indicative of small airways dysfunction.

## Conclusion

The CS exposed workers had higher MMP-12 concentrations than the referents, and a dose-related increase in MMP-12 was observed. The other alterations observed may be related to a down-regulation of pro-inflammatory pathways.

## Data Availability

Data supporting the results of this study are not openly available due to reasons of sensitivity, to protect the confidentiality of the information provided by the participants. Data may be provided by the corresponding author upon reasonable request.
